# Analysis of the rs10046 Polymorphism of Aromatase (CYP19) in Premenopausal Onset of Human Breast Cancer

**DOI:** 10.3390/ijms15010712

**Published:** 2014-01-07

**Authors:** Karin Zins, Maurice Mogg, Christian Schneeberger, Dietmar Abraham, Martin Schreiber

**Affiliations:** 1 Laboratory for Molecular Cellular Biology, Center for Anatomy and Cell Biology, Medical University of Vienna, A-1090 Vienna, Austria; E-Mails: karin.zins@meduniwien.ac.at (K.Z.); dietmar.abraham@meduniwien.ac.at (D.A.); 2 Department of Obstetrics and Gynecology, Medical University of Vienna, Waehringer Guertel 18-20, A-1090 Vienna, Austria; E-Mails: maurice.mogg@gmail.com (M.M.); christian.schneeberger@meduniwien.ac.at (C.S.); 3 Comprehensive Cancer Center (CCC), Medical University of Vienna, A-1090 Vienna, Austria

**Keywords:** breast cancer, CYP19/aromatase, rs10046, rs2236722, SNP

## Abstract

The *CYP19* gene encodes aromatase, an enzyme catalyzing the conversion of androgens to estrogens. Studies analyzing associations between single nucleotide polymorphisms in *CYP19* and breast cancer risk have shown inconsistent results. The rs10046 polymorphism is located in the 3′ untranslated region of the *CYP19* gene, but the influence of this polymorphism on breast cancer risk is unclear. In this study, we investigated the impact of rs10046 SNP on breast cancer risk, age at onset and association with clinical characteristics in an Austrian population of 274 breast cancer patients and 253 controls. The results show that a significantly increased fraction of patients with the TT genotype of rs10046 develop breast cancer under the age of 50 (41.8% of TT patients, compared to 26.6% of C carriers; *p* = 0.018, Chi-square test). No rs10046 genotypes were significantly associated with increased breast cancer risk or patient characteristics other than age at onset. These results suggest that the rs10046 polymorphism in the *CYP19* gene may have an effect on breast cancer susceptibility at an age under 50 in the investigated population.

## 1. Introduction

The hormone estrogen is involved both in the development of the mammary gland, as well as the pathogenesis and progression of breast cancer in both pre- and post-menopausal women [1]. In premenopausal women, the main source of estrogens is the ovaries, whereas in postmenopausal women, estrogen production takes place elsewhere such as in adipose tissue, skin and muscle [2]. Although circulating estrogen concentrations are very low after menopause, peripheral tissues can generate concentrations that are sufficient to stimulate tumor growth. Approximately 80% of breast cancers diagnosed in postmenopausal women are estrogen receptor (ER) and/or progesterone receptor (PR) positive. In this population, the major source of estrogen is the peripheral synthesis of estrone (E1) and estradiol (E2) by the enzyme aromatase [3]. Aromatase is a member of the cytochrome P450 superfamily and is the rate-limiting enzyme in the conversion of androgens into estrogens. Aromatase is a 503-amino acid protein encoded by the *CYP19* gene, which is located at 15q21.2 in humans and contains 10 exons [4,5]. The entire human *CYP19* gene spans over 123 kb of DNA and contains a large 93 kb 5′-flanking region that serves as the regulatory unit of the gene. This regulatory region contains several tissue-specific promoters that are alternatively used in various cell types. Each promoter gives rise to an mRNA with a specific 5′-untranslated region but with an identical coding region and therefore an identical protein regardless of the tissue site of expression [4]. The most proximal promoters are the ovarian-specific promoter II, the I.3 expressed in adipose tissue and breast cancer and the promoter I.6 expressed in bone which are all located within 1 kb of the translation start site. In breast cancer, four promoters (II, I.3, I.7 and I.4) seem to be involved in regulation of aromatase expression [4,6].

Aromatase is expressed in breast tissue, and intratumoral aromatase is the source of local estrogen production in breast tumors [7]. Aromatase inhibitors (AIs), such as anastrozole, constitute an important approach for reducing growth-stimulatory effects of estrogens in estrogen-dependent ER and PR-positive postmenopausal breast cancer patients [7,8]. Because hormone receptor positive breast cancers are largely driven by the estrogen/ER pathway, variation within genes involved in hormone production and regulation is hypothesized to be particularly important.

Several studies found that some polymorphisms in the *CYP19* gene may have effects on breast cancer prognosis depending on menopausal status whereas others were not found to be associated with survival [9–11]. Likewise, studies analyzing *CYP19* polymorphisms and sex hormone levels revealed conflicting results [12–16]. More recently, selected *CYP19* single nucleotide polymorphisms (SNPs) have been investigated for association with therapeutic efficacy and toxicity of AIs. While the genetic variations of *CYP19* rs10459592 and rs4775936 were significantly associated with higher clinical benefit rates of AI in patients with metastatic breast cancer [17], another study showed that the same *CYP19* SNPs were not independently associated with improved AI efficacy in patients with hormone receptor-positive metastatic breast cancer [18]. Several previous studies have investigated polymorphisms on the *CYP19* gene in relation to breast cancer risk, although with conflicting results. It has been suggested that *CYP19* variation may enhance breast cancer development in some women [19], and that the potentially functional *CYP19*_630 3 bp Del/Ins polymorphism and the *CYP19*_681 (TTTA)*n* polymorphism may play a low penetrance role in breast cancer susceptibility in an ethnic specific manner [20]. Other studies, however, observed no significant associations of breast cancer risk with common *CYP19* gene variants [14,21] and differences in estrogen levels caused by genetic variation in *CYP19* were insufficient to contribute detectably to breast cancer [14].

Thus, polymorphisms studied frequently generated inconsistent results. This is also the case for the rs10046 SNP, which is a C/T variation located in the 3′ untranslated region (3′-UTR) of the *CYP19* gene, 19 bp downstream of the amber stop codon in exon 10. Studies indicated that the rs10046 polymorphism was associated with the percentage of HER2-positive tumors, and rs10046 genotypes were associated with an altered disease-free survival (DFS), an effect that appeared to be determined in the subgroup of premenopausal patients [9]. Some studies have linked this polymorphism with breast cancer risk [22], whereas others have shown contradictory results, ranging from no association [23] to age-specific association with breast cancer risk [24]. It has also been shown that the *CYP19* rs10046 polymorphism is associated with breast cancer risk among Chinese women [25]. Another recent meta-analysis showed neither a significant association for rs10046 with breast cancer risk nor association with ethnic subgroups [26].

These divergent results led us to analyze the association of the rs10046 and rs2236722 SNPs in the *CYP19* gene with clinical characteristics of breast cancer. These analyses revealed that SNP rs2236722 was non-polymorphic in our study population, since all patients and controls had the same homozygous genotype. In contrast, rs10046 was polymorphic and we have evaluated its association with breast cancer risk, age at onset and clinical characteristics in a hospital-based case-control study of 276 consecutive breast cancer patients and 255 controls. We found that the TT genotype of the rs10046 SNP is associated with a significantly increased frequency of premenopausal breast cancer onset. However, the TT genotype was not associated with an increased incidence overall and hence the clinical relevance of our finding is presently unclear. The data thus highlights the critical impact of the rs10046 SNP in *CYP19* on breast cancer biology.

## 2. Results and Discussion

### 2.1. *CYP19* rs10046 SNP and Breast Cancer Risk

Two SNPs in the *CYP19* gene were genotyped in a hospital-based case-control study. SNP rs2236722 (Trp39Arg; c.115T > C) was genotyped in 330 subjects (183 cases and 147 controls), which all exhibited the same genotype (TT). Accordingly, this SNP was considered as non-polymorphic in the study population, and was not analyzed further. SNP rs10046 was genotyped in 527 individuals (274 consecutive breast cancer patients and 253 female control subjects). Table 1 shows the clinical characteristics of the study population, together with the frequency of the rs10046 genotypes in the study population and subpopulations. Both the control population (*p* = 0.27) and breast cancer patients (*p* = 0.61) were in Hardy-Weinberg equilibrium. The frequency of the minor C-allele was 49.6% in patients and 48.6% in controls. The fraction of patients with the TT genotype tended, although not with statistical significance, to be increased in patient subgroups associated with advanced cancer stage (28.0% stages II, III and IV *vs*. 19.7% stage 0 and I patients) and lymph node-positive cancer (30.9% lymph node-positive *vs*. 18.9% lymph node-negative patients; Table 1). Moreover, the CC genotype was under-represented in HER2 positive patients (13.0%) compared to HER2 negative patients (26.3%; Table 1), consistent with a previous report [9].

To determine odds ratios and 95% confidence intervals for breast cancer risk, various comparisons of rs10046 genotypes as well as C *vs*. T alleles were analyzed. These comparisons revealed odds ratios between 0.97 and 1.13, which were not significantly different from unity (Table 2). Thus, none of the investigated genotypes or alleles was *per se* associated with increased breast cancer risk.

A list of odds ratios for the different genotypes in specific breast cancer subpopulations is shown in Table 3. Specifically, the odds ratios of specific breast cancer subpopulations for CC *vs*. TT and TC *vs*. TT genotypes, as well as C *vs*. T alleles were evaluated. The odds ratio for patients with breast cancer under age 50 for TC *vs*. TT subjects was 0.59 (95% CI, 0.33–1.04) and for CC *vs*. TT carriers 0.76 (95% CI, 0.39–1.52). Thus, the TT genotype tended to be associated with an increased breast cancer risk in this age group, although these differences were not significant (Table 3). A similar trend was observed in pre-menopausal patients, a subpopulation with a large overlap with patients under the age of 50 (Table 3). Furthermore, a trend for a non-significantly-increased risk associated with the T-allele, as indicated by odds ratios <1 in Table 3 was observed in HER2 positive and p53 positive patients (Table 3). Conversely, a trend for increased breast cancer risk associated with the C allele, as indicated by odds ratios >1 in Table 3 was observed in patients with tumors larger than 2 cm (pT2–pT4) and in patients without lymph node metastases (pN0). However, none of these associations reached statistical significance at the *p* < 0.05 level (Table 3).

### 2.1.1. SNP rs10046 and Age at Breast Cancer Onset

**Figure 1.**
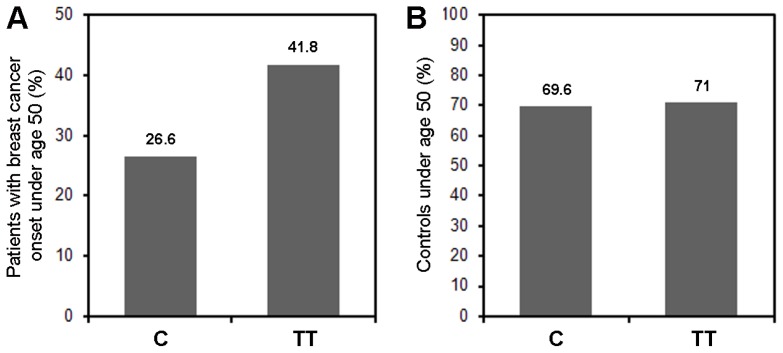
Breast cancer patients with the TT genotype exhibit an increased frequency of early onset. (**A**) percentage of breast cancer patients under the age of 50 with the TT genotype (TT) *vs*. the CC or TC genotype (C-carriers, C). *p* = 0.018, Chi-square test; (**B**) controls under the age of 50 at the time of recruitment are shown for comparison (*p* = 0.84).

**Figure 2.**
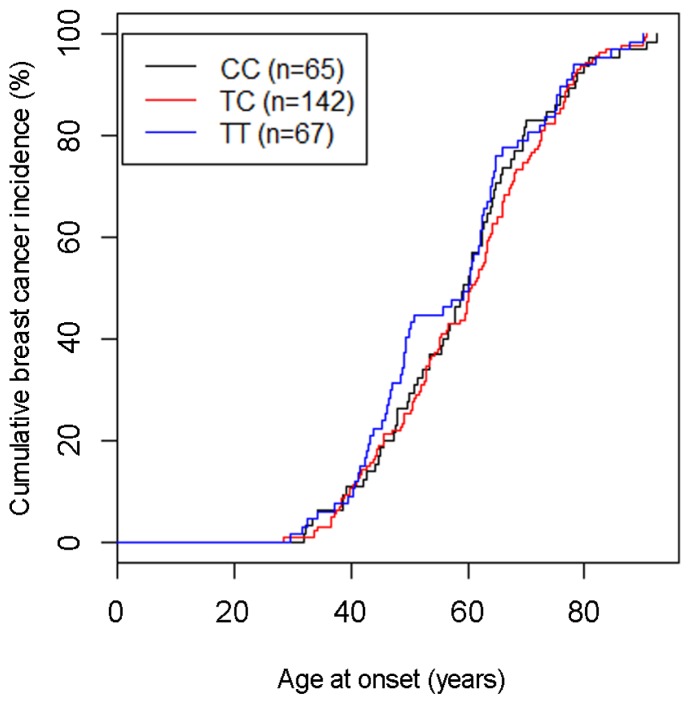
Curves of the cumulative breast cancer incidence at the indicated ages at onset of patients with genotypes CC, TC and TT.

In order to explore the potential impact of the CC, TC and TT genotypes on breast cancer onset, we analyzed the association of these genotypes with age at breast cancer diagnosis. We found that 28/67 TT patients (41.8%), 36/142 TC patients (25.4%) and 19/65 CC patients (29.2%) developed breast cancer at an age below 50. Thus, 41.8% of patients with the TT genotype, but only 26.6% of C carriers (55/207) were diagnosed with breast cancer at an age younger than 50 (*p* = 0.018, Chi-square test; Figure 1). Comparison of the cumulative breast cancer incidence of all three genotypes also revealed differences between the TT genotype and the two other genotypes (Figure 2). The curve of cumulative incidence of TT patients exhibited a considerably steeper slope than the TC and CC genotypes in an age group between 40 and 50, whereas the three curves aligned again at higher ages-at-onset. The resulting kink in the graph of patients with the TT genotype indicates a higher breast cancer incidence in premenopausal patients (Figure 2; Table 3). However, the effect size is rather small and it is presently unclear whether it is clinically meaningful. A plateau phase around menopause (age 50–55) was observed in TT patients showing that there was no increased risk of developing cancer during this phase. In postmenopausal patients, no significant differences in breast cancer rates were observed between the three genotypes (Figure 2).

### 2.2. Discussion

Circulating estradiol levels are genetically controlled and have been related to breast cancer risk. Since genetic variation in the *CYP19* gene contributes to variance in circulating hormone levels, it is tempting to speculate that genetic polymorphisms in this gene such as the rs10046 SNP, a T-C variant in the 3′ untranslated region, are potential candidates to have an impact on breast cancer risk [12]. The *CYP19* polymorphism rs10046 has been extensively studied in different populations. A Chinese study provided evidence that the *CYP19* rs10046 polymorphism is associated with breast cancer risk among Chinese women [25] and results from a study in a Spanish population also revealed an association between rs10046 and breast cancer risk. In this study, the carriers of at least one C allele had an increased risk of developing breast cancer [26], which is in agreement with other studies, where the frequency of the C allele is higher in cases *vs.* controls [12,19]. In contrast, Kristensen *et al*. found that the rs10046 T-allele of the *CYP19* gene is associated with a “high activity” phenotype and that the TT genotype of this polymorphism was associated with increased breast cancer risk [22]. Similar to that study, our data suggest that the TT genotype has a tendency to be overrepresented in patient subgroups associated with advanced cancer stage and lymph node-positive cancer (Tables 1 and 3).

However, our data reveal that none of the investigated genotypes was *per se* associated with a significantly increased breast cancer risk. Likewise, other authors found no association between rs10046 and breast cancer risk [23]. A recent meta-analysis of 20,098 subjects showed neither a significant association for rs10046 with breast cancer risk nor associations with ethnic subgroups [26]. The same study indicated no existence of a trend for the rs10046 genetic variants between cases and controls. The authors discuss different populations, geographical areas and variable number of samples used in the studies as possible reasons for these inconsistent results [26]. Thus, it seems to be clear that further studies are necessary prior to drawing any conclusions on the possible association of rs10046 with breast cancer risk.

In this regard, we have performed a study in an Austrian population including only women of Caucasian background from the same geographical area. Our findings in the investigated Austrian population add a new, to date unidentified aspect of the potential impact of the rs10046 SNP on breast cancer. Our data reveal that a significantly higher fraction of patients with the TT genotype exhibited a younger age at breast cancer onset. Though 41.8% of the TT patients were diagnosed with breast cancer at an age under 50, only 26.6% of C carriers (patients with the CC or TC genotype) were. Moreover, at an age between 40 and 50, TT patients exhibited a considerably steeper increase in the cumulative breast cancer incidence compared to patients with the TC and CC genotypes. However, the incidence rates of the three rs10046 genotypes aligned again at an age above approximately 50–55, which is the age at which most women undergo menopause. Accordingly, the mean age at onset was not significantly different overall, and hence it is presently unclear whether this effect is clinically meaningful. Similar to the patients under 50 years of age, there was an increased frequency of TT patients compared to C carriers in premenopausal patients, but this difference was not significant (*p* = 0.098, Chi-square test). An explanation may be that the age at onset, but not the menopausal status, is known for all patients and controls in the study population (see Table 1). Specifically, the study population included 83 patients with an age at onset below 50, but only 63 with a confirmed premenopausal status, thus reducing the statistical power of the analyses according to menopausal status. Collectively, our data indicate a higher fraction of breast cancer onset in premenopausal patients with the TT genotype, which is counterbalanced during and/or after menopause.

It has been claimed that rs10046 is related to the levels of estradiol and the estradiol: testosterone ratio in normal postmenopausal women [12], a factor important for the development of breast cancer [1] and for the use of aromatase inhibitors in postmenopausal breast cancer patients [27]. A significant association of aromatase SNPs and haplotypes with circulating estrogen levels among postmenopausal women has been found by Haiman *et al*. [14]. Presence of the rs10046 SNP C allele is associated with reduced estradiol levels [12]. Moreover, Kristensen *et al*. [22] reported that the C allele is associated with lower levels of *CYP19* mRNA in tumors. Thus, it has been suggested that the C allele generates less aromatase enzyme than the T allele and hence confers reduced overall enzyme activity [12]. From these data one might conclude that the rs10046 polymorphism contributes to levels of circulating estradiol. Unfortunately, serum samples were not available for the determination of estradiol, which could be a limitation of the study by Kristensen *et al*. [22], as it could have provided more information on the role of *CYP19* in premenopausal breast cancer patients.

However, based on measurements of estradiol levels in the same individual at different times, it has been reported that approximately 50% of the variance in estradiol levels is essentially random fluctuation [28]. Based on this assumption and on their measured mean values of estradiol associated with the rs10046 TC and TT genotypes, Dunning *et al*. [12] have predicted odds ratios of 1.10 and 1.03 in women with the TT and TC genotypes, respectively. They calculated that a study of approximately 34,000 cases and a similar number of matched controls would be required to detect such a moderate risk with sufficient statistical power, which clearly would be a limitation of measurements of circulating estradiol levels in smaller studies [12]. Thus, larger studies with repeated lifetime measurements would be necessary to draw conclusions on the true relationship between estradiol levels and breast cancer risk associated with rs10046. In addition, it still remains unclear how this SNP might affect estrogen levels [26,29]. In the absence of a mechanistic explanation, a strong linkage disequilibrium with other polymorphisms remains possible [26].

The rs10046 CC genotype has also been reported to be associated with a lower percentage of HER2-positive tumors [9], in agreement with our results. Interestingly, this study also showed that the CC genotype of rs10046 was associated with a better disease-free survival in pre-menopausal, but not post-menopausal patients [9]. Likewise, our data suggest an association of the TT genotype with a higher relative breast cancer incidence in premenopausal patients. Thus, the rs10046 genotypes of *CYP19* may influence tumor onset and characteristics for premenopausal breast cancer patients.

## 3. Experimental Section

### 3.1. Study Population

The study population has been described in detail in [30,31]. The clinical and histopathological characteristics of the group are shown in Table 1. Two hundred and seventy-six consecutive female breast cancer patients and 255 controls (patients with benign gynecological lesions and healthy females) of Caucasian background were enrolled between 2002 and 2004 at the Department of Obstetrics and Gynecology, Medical University of Vienna (MUV), Vienna, Austria. This study was approved by the institutional review board of the MUV and written informed consent was obtained from all participants. rs2236722 genotypes were determined in 330 subjects (183 cases and 147 controls), which all exhibited the same genotype. Thus, this SNP was not analyzed further and genotyping of the remaining 201 subjects was not attempted. Determination of the rs10046 genotype was unsuccessful for 2 patients and 2 controls, and all analyses of this SNP were based on the remaining 527 subjects (Table 1).

### 3.2. DNA Isolation and Genotyping

Genomic DNA was extracted from blood samples with the QIAamp DNA Blood Midi kit (Qiagen, Venlo, The Netherlands) following the manufacturer’s instructions. Genotyping of SNP rs10046 (*CYP19* E10 c.+19C > T; located in Exon 10, 19 bp downstream from the amber stop codon) and rs2236722 (Trp39Arg; c.115T > C) was performed by TaqMan with allele-specific, fluorescently labeled probes following the manufacturer’s instructions (Applied Biosystems, Brunn/Gebirge, Austria; Assay-ID # C___8234731_30 and C__15954948_40, respectively). Forty nano grams of genomic DNA were used per reaction in a total reaction volume of 10 μL. Alternatively, genotyping of SNP rs10046 was performed by two separate allele-specific conventional PCR reactions followed by agarose gel electrophoresis with the following primers: forward, 5′-ATATTCTGGCAACTGTCTG-3′ and reverse, 5′-GAGAAATGCTCCAGAGTG-3′ to detect the C-allele; forward, 5′-AAGGCTGGTCAGTACCT-3′ and reverse, 5′-GAGGATGACACTATTGGC-3′ to detect the T-allele. Fifty-one samples were genotyped with both methods, with 96.1% concordant results.

### 3.3. Statistical Analysis

Statistical analyses were performed with R, an open-source language and environment for statistical computing [32]. Potential deviations of the study population from Hardy-Weinberg equilibrium were assessed with Chi-square tests with Yates’ continuity correction. Confidence intervals given are 95% mid-*p* exact confidence intervals, *i.e.*, considering all possible configurations of the contingency table that are more extreme than the observed configuration, and half the configurations that are equivalent to the observed one. Likewise, *p*-values shown in Table 2 are mid-*p* two-tailed exact *p*-values. Associations between the three *CYP19* rs10046 genotypes and clinical or histopathological characteristics were evaluated with Chi-square tests. Since we consider the subgroup analyses reported in Tables 1 and 3 as exploratory, we did not correct for multiple testing, following a previous recommendation [33].

## 4. Conclusions

In conclusion, despite the restriction of this study to a limited population, the results suggest that the TT genotype of the rs10046 polymorphism in the *CYP19* gene is associated with a higher relative breast cancer incidence in premenopausal patients. We found no evidence for a significant association of this genotype with breast cancer risk in other patient populations. These results suggest that the TT polymorphism gene may have an effect on breast cancer susceptibility in premenopausal patients of the investigated ethnic subpopulation. Further studies are necessary to clarify the possible influence of the rs10046 *CYP19* polymorphism on circulating estradiol levels in premenopausal patients. The present case-control study can only assess relative incidence rates. Prospective studies to assess the impact of the TT genotype of rs10046 SNP on absolute breast cancer incidence rates under age 50 are warranted.

## Figures and Tables

**Table 1.** Clinical characteristics of the study population, and frequency of the *CYP19* rs10046 genotypes in the indicated subpopulations.

		Total	CC	TC	TT	*p*-value
All subjects		527	120 (22.8%)	278 (52.8%)	129 (24.5%)	
Patients		274	65 (23.7%)	142 (51.8%)	67 (24.5%)	
Controls		253	55 (21.7%)	136 (53.8%)	62 (24.5%)	

**Patient subgroups**						

Age (years)	<50	83	19 (22.9%)	36 (43.4%)	28 (33.7%)	0.053
	≥50	191	46 (24.1%)	106 (55.5%)	39 (20.4%)	

Menopausal status	pre	63	13 (20.6%)	29 (46.0%)	21 (33.3%)	0.249
	post	176	40 (22.7%)	96 (54.5%)	40 (22.7%)	
	na	35	12 (34.3%)	17 (48.6%)	6 (17.1%)	

Tumor size	pT1	136	28 (20.6%)	72 (52.9%)	36 (26.5%)	0.339
	pT2–4	67	20 (29.9%)	32 (47.8%)	15 (22.4%)	
	other, na	71	17 (23.9%)	38 (53.5%)	16 (22.5%)	

Tumor type	ductal	153	41 (26.8%)	76 (49.7%)	36 (23.5%)	0.996
	lobular	48	13 (27.1%)	24 (50.0%)	11 (22.9%)	
	other, na	73	11 (15.1%)	42 (57.5%)	20 (27.4%)	

Stage	0 or I	117	27 (23.1%)	67 (57.3%)	23 (19.7%)	0.193
	II–IV	93	25 (26.9%)	42 (45.2%)	26 (28.0%)	
	other, na	64	13 (20.3%)	33 (51.6%)	18 (28.1%)	

Grade	pG1–2	161	39 (24.2%)	82 (50.9%)	40 (24.8%)	0.982
	pG3	92	23 (25.0%)	47 (51.1%)	22 (23.9%)	
	na	21	3 (14.3%)	13 (61.9%)	5 (23.8%)	

Lymph node status	pN0	148	38 (25.7%)	82 (55.4%)	28 (18.9%)	0.181
	pN+	55	13 (23.6%)	25 (45.5%)	17 (30.9%)	
	na	69	15 (21.7%)	30 (43.5%)	24 (34.8%)	

ER status	pos	202	48 (23.8%)	100 (49.5%)	54 (26.7%)	0.690
	neg	60	14 (23.3%)	33 (55.0%)	13 (21.7%)	
	na	12	3 (25.0%)	9 (75.0%)	0 (0.0%)	

PR status	pos	142	31 (21.8%)	76 (53.5%)	35 (24.6%)	0.604
	neg	120	31 (25.8%)	57 (47.5%)	32 (26.7%)	
	na	12	3 (25.0%)	9 (75.0%)	0 (0.0%)	

HER2 status	pos	54	7 (13.0%)	31 (57.4%)	16 (29.6%)	0.119
	neg	205	54 (26.3%)	101 (49.3%)	50 (24.4%)	
	na	15	4 (26.7%)	10 (66.7%)	1 (6.7%)	

Numbers of patients in each of the indicated subgroups are shown; Numbers in parentheses indicate the fraction of patients in percent with the corresponding genotypes CC, TC and TT, respectively; na, status not available; ER, estrogen receptor; PR, progesterone receptor; *p*-values of subgroup comparisons were calculated with chi-square tests.

**Table 2.** Odds ratios, 95% confidence intervals and *p*-values for the association of *CYP19* rs10046 genotypes and alleles with breast cancer risk.

Genotypes	OR	95% CI	*p*-value
CC *vs*. TT	1.09	0.66–1.80	0.752
CC *vs*. TC	1.13	0.74–1.74	0.549
TC *vs*. TT	0.97	0.64–1.47	0.873
CC + TC *vs*. TT	1.00	0.67–1.49	0.989
CC *vs*. TC + TT	1.12	0.74–1.68	0.587
C *vs.* T	1.04	0.81–1.34	0.734

Analyses of breast cancer cases *vs.* controls of the indicated genotypes are shown; OR, odds ratios; 95% CI, 95% confidence intervals.

**Table 3.** Odds ratios, 95% confidence intervals and *p*-values for association of the *CYP19* rs10046 polymorphism with breast cancer risk in the indicated patient subgroups.

	Subgroup	No. of cases (%)		CC *vs*. TT			TC *vs*. TT			C *vs.* T		
		
				OR	95% CI	*p*-value	OR	95% CI	*p*-value	OR	95% CI	*p*-value
Age (years) 1	<50	83	30.3%	0.76	(0.39–1.52)	0.44	0.59	(0.33–1.04)	0.06	0.85	(0.59–1.21)	0.36
	≥50	191	69.7%	1.33	(0.76–2.34)	0.32	1.24	(0.77–2.00)	0.37	1.14	(0.87–1.48)	0.34

Menopausal status	pre	63	26.4%	0.70	(0.32–1.52)	0.38	0.63	(0.33–1.19)	0.16	0.81	(0.54–1.21)	0.31
	post	176	73.6%	1.13	(0.64–1.99)	0.72	1.09	(0.68–1.76)	0.76	1.06	(0.80–1.41)	0.68

Tumor type	ductal	153	76.1%	1.28	(0.72–2.28)	0.42	0.96	(0.59–1.58)	0.85	1.13	(0.85–1.52)	0.39
	lobular	48	23.9%	1.33	(0.55–3.22)	0.58	0.99	(0.46–2.16)	0.92	1.16	(0.74–1.82)	0.52

Tumor size	pT1	136	67.0%	0.88	(0.47–1.62)	0.70	0.91	(0.55–1.50)	0.75	0.93	(0.69–1.27)	0.67
	pT2–4	67	33.0%	1.50	(0.70–3.22)	0.29	0.97	(0.49–1.93)	0.93	1.24	(0.84–1.84)	0.28

Stage	0 or I	117	55.7%	1.32	(0.68–2.57)	0.45	1.33	(0.76–2.33)	0.30	1.15	(0.83–1.59)	0.41
	II–IV	93	44.3%	1.08	(0.56–2.09)	0.80	0.74	(0.41–1.31)	0.27	1.04	(0.74–1.46)	0.84

Grade	pG1–2	161	63.6%	1.10	(0.62–1.95)	0.72	0.93	(0.58–1.51)	0.76	1.05	(0.79–1.39)	0.76
	pG3	92	36.4%	1.18	(0.59–2.35)	0.66	0.97	(0.54–1.75)	0.94	1.09	(0.77–1.54)	0.64

Nodal status	pN0	148	72.9%	1.53	(0.83–2.83)	0.19	1.34	(0.79–2.27)	0.27	1.21	(0.91–1.61)	0.20
	pN+	55	27.1%	0.86	(0.38–1.93)	0.76	0.67	(0.34–1.33)	0.24	0.91	(0.60–1.39)	0.66

ER status	pos	202	77.1%	1.00	(0.59–1.71)	0.95	0.84	(0.54–1.32)	0.46	1.00	(0.76–1.30)	0.98
	neg	60	22.9%	1.21	(0.53–2.81)	0.60	1.16	(0.57–2.35)	0.66	1.10	(0.73–1.67)	0.65

PR status	pos	142	54.2%	1.00	(0.55–1.83)	0.94	0.99	(0.60–1.63)	0.95	1.00	(0.74–1.35)	0.99
	neg	120	45.8%	1.09	(0.59–2.02)	0.82	0.81	(0.48–1.38)	0.46	1.04	(0.76–1.42)	0.80

HER2 status	pos	54	20.8%	0.49	(0.19–1.29)	0.14	0.88	(0.45–1.73)	0.67	0.73	(0.47–1.14)	0.17
	neg	205	79.2%	1.22	(0.72–2.07)	0.46	0.92	(0.59–1.45)	0.69	1.10	(0.85–1.44)	0.47

Ki67 status	>10%	105	48.2%	1.08	(0.22–2.13)	0.80	1.10	(0.63–1.93)	0.72	1.04	(0.74–1.46)	0.82
	≤10%	113	51.8%	1.21	(0.65–2.25)	0.58	0.83	(0.48–1.43)	0.53	1.10	(0.80–1.51)	0.56

p53 status	pos	58	22.7%	0.65	(0.29–1.49)	0.36	0.67	(0.35–1.29)	0.20	0.79	(0.52–1.20)	0.27
	neg	197	77.3%	1.18	(0.68–2.03)	0.54	1.02	(0.64–1.62)	0.95	1.08	(0.83–1.42)	0.56

ER, estrogen receptor; PR, progesterone receptor; 95% CI, 95% confidence intervals;

1 patients aged under 50 years or ≥50 years at diagnosis were compared to control subjects of any age.
